# Cyclical and seasonal variations in the incidence of type 1 diabetes mellitus between 1985 and 2016 in Bauru, São Paulo, Brazil

**DOI:** 10.20945/2359-4292-2025-0156

**Published:** 2025-09-28

**Authors:** Gabriel Araújo Felinto Medeiros, Lucas Casagrande Passoni Lopes, Carlos Antonio Negrato

**Affiliations:** 1 Faculdade de Medicina de Bauru, Universidade de São Paulo, Bauru, SP, Brasil

**Keywords:** Type 1 diabetes mellitus, epidemiology, periodicity

## Abstract

**Objective:**

To evaluate cyclical and seasonal variation in the incidence of type 1 diabetes mellitus (T1DM) from 1985 to 2016 in Bauru, São Paulo, Brazil.

**Subjects and methods:**

This was a retrospective longitudinal study. Clinical data were collected for individuals known to have T1DM, who aged from 0-14 years, residing in Bauru, São Paulo State, and followed at a local endocrinology clinic from 1985 to 2016. Incidence rates were calculated annually and grouped into quadrennial intervals. Trends were analyzed using Joinpoint Regression to estimate annual percentage changes. Poisson regression models assessed cyclical and seasonal patterns over various periods (3- to 7.5-year cycles). Seasonal variation was evaluated using the Akaike Information Criterion and chi-square likelihood ratios to assess model fit.

**Results:**

Among the 298 included patients, the mean annual incidence was 12.1 per 100,000 person-years (95% CI: 10.7-13.4), with an average annual increase of 2.77% (95% CI: 1.3-4.3%). A significant cyclical variation of 18% every 7.5 years was observed, with girls exhibiting a 22.9% variation every 5 years. No cyclical pattern was identified for boys. Seasonal analysis revealed higher amplitudes among girls (±26.4%) and in the 5-9.99-year age group (±26.2%), predominantly during colder months.

**Conclusion:**

T1DM cyclical variations with a 7.5-year cycle were observed, with girls showing a pronounced variation and a distinct 5-year cycle. Seasonal variations were found among girls, particularly in the 5-9.99-year age group. Outbreaks of H1N1 and dengue, along with the lowest temperatures, coincided with higher incidence rates, aligning with the 7.5-year cycles. Targeted health policies are needed to mitigate the impact of these factors, supporting surveillance, early diagnosis, and preventive strategies for T1DM.

## INTRODUCTION

Insufficient endogenous insulin secretion is the hallmark of type 1 diabetes mellitus (T1DM) (^1^). Approximately 90% of cases arise from autoimmune phenomena that lead to pancreatic β-cell destruction, while the remaining 10% are autoantibody negative (^1^). This condition has an impactful incidence, mostly among young individuals (^1^). However, these rates vary by country, within countries, and over time (^1^).

Substantial cyclical variation in T1DM incidence has been reported over the years (^2^-^6^). A 4-year cycle was identified in studies conducted in England and Italy between 1978-1990 and 1989-2021, respectively (^2^,^3^). A 25-year prospective study (1989-2013) covering 26 European countries also found 4-year cycles (^4^). Similarly, a study from Western Australia (1985-2010) reported 5-year cycles (^5^), and another British study (1990-2010) identified 6-year cycles (^6^).

Environmental factors are believed to play key roles in the cyclical patterns observed in T1DM onset (^7^,^8^). Infections and temperature fluctuations may interact with specific genes and trigger T1DM development in genetically predisposed individuals (^7^,^8^). As a result, recurring viral epidemics and climate variability may precede emergent cycles in T1DM incidence (^4^,^5^,^7^,^8^).

According to the International Diabetes Federation estimates, nearly 100,000 Brazilians under 19 years of age had T1DM in 2021, and approximately 10,000 new cases are diagnosed each year in the country (^9^). However, few studies have investigated the epidemiology of T1DM in Brazil, and its cyclical and seasonal variations remain unknown (^10^,^11^).

Therefore, this study aimed to evaluate the cyclical and seasonal variations in the incidence of T1DM from 1985 to 2016 in Bauru, São Paulo, Brazil.

## SUBJECTS AND METHODS

### Study characterization

This retrospective longitudinal study was performed upon previous evaluations conducted in Bauru, which assessed T1DM incidence from 1985 to 2016, with a new focus on the potential cyclical and seasonal variations in these rates (^10^,^11^).

### Local identification

Bauru is the most populous city in the central-western region of São Paulo, the wealthiest and most populous Brazilian state. It also serves as a strategic hub for the movement of goods, services, and people throughout São Paulo and the broader country.

Founded 129 years ago, the city is located at a latitude of 22º18’54”N and a longitude of 49º03’39”W (^12^). It lies at an average elevation of 526 meters above the sea level and has a tropical high-altitude climate, and spans 667,658 square kilometers. Its population stands at 379,146 residents, with 338,891 people living in urban areas and 5,148 in rural zones (^12^). Of the population, 195,826 are women (51.65%) and 183,320 are men (48.35%) (^12^). Additionally, 65,931 inhabitants (17.39%) are aged 0-14 years, 265,517 (70.20%) are 15-64 years old, and 47,698 people (12.59%) are over 65 years old (^12^). The city has a Human Development Index (HDI) of 0.801 (^12^).

### Data setting

A primary database was compiled through collaboration with all endocrinologists, pediatricians, general practitioners, and all public and private schools located in Bauru from 1985 to 2016, by the capture-recapture method (^13^). This database was previously used in two earlier studies evaluating T1DM incidence in Bauru. Of the initial 302 individuals, four were excluded due to incomplete data for this specific analysis, resulting in 298 participants.

Data collected included sex, age at diagnosis, family income, ethnicity, and the year of T1DM diagnosis. These diagnoses were made by physicians following criteria established by the American Diabetes Association and Brazilian Diabetes Society. The codes included to consider the T1DM outcome were E250x from the 9th International Classification of Diseases (ICD-9) up to 1995, and E10 from the 10th International Classification of Diseases (ICD-10) from 1996 onwards. Data from 2017 onward were excluded due to incompleteness in the primary database.

Annual population estimates were provided by the Brazilian Institute of Geography and Statistics (IBGE), available on the Datasus website (^14^). The data were collected between May 31st and September 17th, 2024, and then organized into Excel^®^ spreadsheets.

### Statistical analysis

Initially, the study population was stratified annually by sex and age. Subsequently, incidence rates per 100,000 person-years and confidence intervals were calculated assuming a Poisson distribution. Data were grouped into eight quadrennial periods (1985-1988, 1989-1992, 1993-1996, 1997-2000, 2001-2004, 2005-2008, 2009-2012, and 2013-2016) for descriptive analysis.

### Joinpoint regression

Cyclical variation was assessed by two distinct methods. First, a time series of age-adjusted rates was analyzed using segmented linear regression. Annual percentage change (APC) and average annual percentage change (AAPC) were estimated with 95% confidence intervals (95%CI); the significance level was set at 5%. A positive APC indicates an increasing trend, while a negative APC denotes a decreasing trend. Conversely, a p-value ≥ 0.05 suggests a stationary trend and was not considered statistically significant. Models incorporating 0 to 9 joinpoints were tested. This analysis was conducted using Joinpoint Regression software, version 5.0.2 (^15^).

### Poisson regression

Secondly, Poisson regression was applied to analyze incidence rates by calendar year, sex, and age group at diagnosis (0-4.99, 5-9.99, and 10-14.99 years) and to estimate temporal trends. To explore nonlinear variation over the studied period, sine and cosine functions were included in Poisson regression models for 3-, 4-, 5-, 6-, 7- and 7.5-year cycles. Additionally, to assess monthly variation over the 384 months of the 32-year period, the same functions and models were applied to test for a 12-month cycle. The sinusoidal functions were obtained through transformation for each analyzed period. Model fit was evaluated using the Akaike Information Criterion (AIC) and chi-square likelihood ratio tests. A significance threshold of 5% (p-value < 0.05) was adopted. All analyses were conducted using IBM Statistical Package for Social Sciences (SPSS), version 25.0, and graphs were generated in R, version 4.3.1, using the ggplot2 package.

## RESULTS

Over the entire period, 302 cases of T1DM were recorded. Four cases had incomplete data; the remaining 298 were included, with 154 cases occurring in girls and 144 in boys. Among all cases, 60 occurred in children aged 0-4 years, 113 in those aged 5-9 years, and 125 among those aged 10-14 years. Most individuals had low family income (53.36%) and were White (80.20%), and the average age at diagnosis was 8.72, ranging from 0.5 to 14.92. The complete sociodemographic characterization of the study population by quadrennium is shown in **Table 1**.

**Table 1:** Sociodemographic characteristics individuals diagnosed with type 1 diabetes mellitus in Bauru between 1985-2016

Variable (Count + N% OR Mean + Range)		Quadrennial periods								Total
		1985-1988	1989-1992	1993-1996	1997-2000	2001-2004	2005-2008	2009-2012	2013-2016	
Sex	Male	4 (1.34)	13 (4.36)	16 (5.37)	23 (7.72)	21 (7.05)	25 (8.39)	20 (6.71)	20 (6.71)	142 (47.65)
	Female	12 (4.03)	18 (6.04)	11 (3.69)	20 (6.71)	22 (7.38)	24 (8.05)	25 (8.39)	24 (8.05)	156 (52.35)
Age		7.24 (2.16-12.6)	8.10 (1.7-14.3)	7.88 (1.0-14.42)	8.85 (1.83-14.82)	8.60 (2.0-14.67)	9.65 (3.16-14.83)	8.95 (0.83-14.92)	8.84 (0.5-14.92)	8.72 (0.5-14.92)
Family Income	Low	7 (2.34)	13 (4.36)	12 (4.03)	21 (7.05)	23 (7.72)	24 (8.05)	31 (10.40)	28 (9.39)	159 (53.36)
	Medium	6 (2.01)	12 (4.03)	11 (3.69)	18 (6.04)	18 (6.04)	19 (6.38)	12 (4.03)	13 (4.36)	109 (36.58)
	High	3 (1.00)	6 (2.01)	4 (1.34)	4 (1.34)	2 (0.67)	6 (2.01)	2 (0.67)	3 (1.00)	30 (10.06)
Ethnicity	White	14 (4.69)	23 (7.72)	20 (6.71)	39 (13.08)	34 (11.41)	41 (13.76)	35 (11.74)	33 (11.07)	239 (80.20)
	Brown	2 (0.67)	7 (2.34)	5 (1.68)	3 (1.00)	7 (2.34)	6 (2.01)	9 (3.02)	9 (3.02)	48 (16.10)
	Black	- (0.00)	1 (0.34)	3 (1.00)	1 (0.34)	2 (0.67)	1 (0.34)	1 (0.34)	2 (0.67)	11 (3.70)

**Figure 1:**
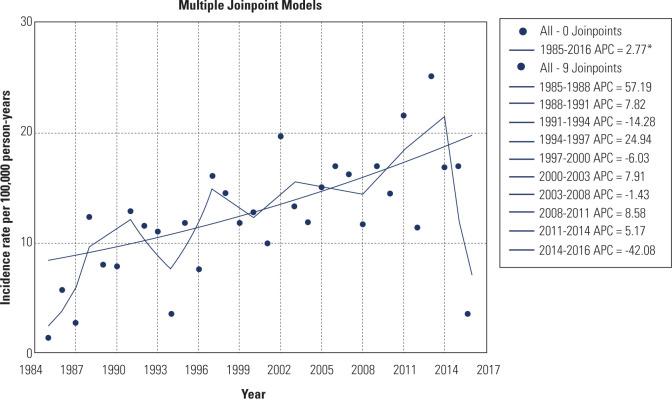
Joinpoint model of type 1 diabetes mellitus incidence over the evaluated period.

The lowest crude and adjusted incidence of T1DM occurred during the 1985–1988 quadrennium, and the highest values were recorded in the 2005-2008 and 2009-2012 periods, respectively. Annual incidence rates are presented in **Table 2**. From 1985 to 2016, the mean incidence rate was 12.1 per 100,000 person-years (95% CI: 10.7-13.4), ranging from 1.5 per 100,000 in 1985 to 25.1 per 100,000 person-years in 2013. The incidence increased by an average of 2.77% per year (95% CI: 1.3-4.3%), as shown in **Figure 1**.

**Table 2:** Incidence rates of type 1 diabetes mellitus over the evaluated period

Period	Count	People-years	Crude incidence			Age-adjusted Incidence		
			Rate	95% CI		Rate	95% CI	
1985-1988	16	280453	5.70	3.55	7.86	5.69	3.71	7.66
1989-1992	31	305603	10.14	9.19	11.09	10.17	7.63	12.70
1993-1996	27	326565	8.26	7.33	9.81	8.27	6.06	10.48
1997-2000	43	325623	13.20	12.85	13.55	13.24	10.44	16.04
2001-2004	43	327298	13.13	11.82	14.45	13.18	10.39	15.96
2005-2008	49	317009	15.45	14.57	16.34	15.98	12.80	19.16
2009-2012	45	285206	15.78	14.34	17.21	15.79	12.52	19.05
2013-2016	44	283945	15.49	12.73	18.25	15.74	12.44	19.03

**Figure 2:**
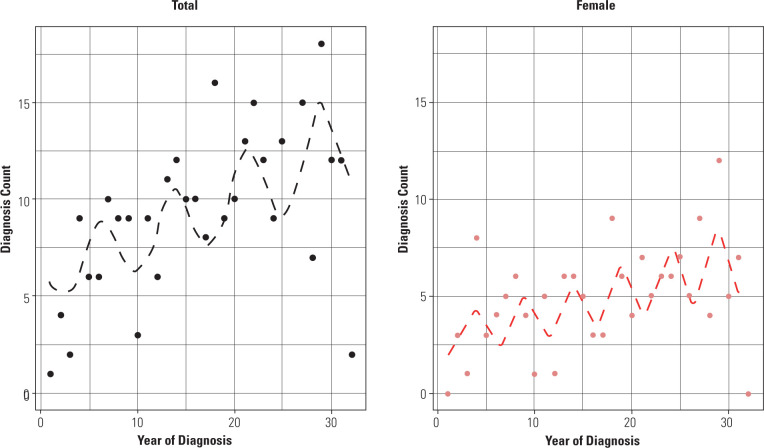
Cyclical variation of type 1 diabetes mellitus diagnosis in Bauru, São Paulo, Brazil, from 1985 through 2016, for females and for both sexes.

A sinusoidal cyclical variation of 18% was observed in the total T1DM count (0.7%-38.1%; p-value < 0.05), with the best model fit corresponding to a 7.5-year cycle (AIC = 179.779). Among girls, a 22.9% variation (3.5%-28.3%; p-value < 0.05) was found, with a best-fit 5-year cycle (AIC = 152.017), as shown in **Figure 2**. Cyclical patterns could not be observed among boys.

Aggregated monthly data over 32 years showed greater seasonal variation among girls (amplitude ± 26.4%) compared with boys (±19.5%). Seasonal variation in girls followed a sinusoidal pattern (p-value < 0.05), whereas no pattern could be identified among boys. Seasonal variation was evaluated in each age-group, with larger amplitudes being observed in the 5-9.99-year group (±26.2%) followed by 10-14 years (±22.1%) and 0-4 years (±20.8%). The 5-9.99-year age group was the only group exhibiting a sinusoidal pattern (p-value < 0.05), as shown in **Table 3**.

**Table 3:** Annual and monthly type 1 diabetes mellitus variations according to Poisson models

Group	Cycle	Variation	95%CI	Sine p-value	Cosine p-value
Total	7.5y	±18%	0.7%-38.1%	0.041	0.118
Female	5y	±22.9%	3.5%-28.3%	0.328	0.023
Male	12m	±19.5%	-	0.142	0.069
Female	12m	±26.4%	-	0.237	0.008
Age 0-4	12m	±20.8%	-	0.383	0.178
Age 5-9	12m	±26.2%	-	0.076	0.025
Age 10-14	12m	±22.1%	-	0.417	0.069

**Figure 3:**
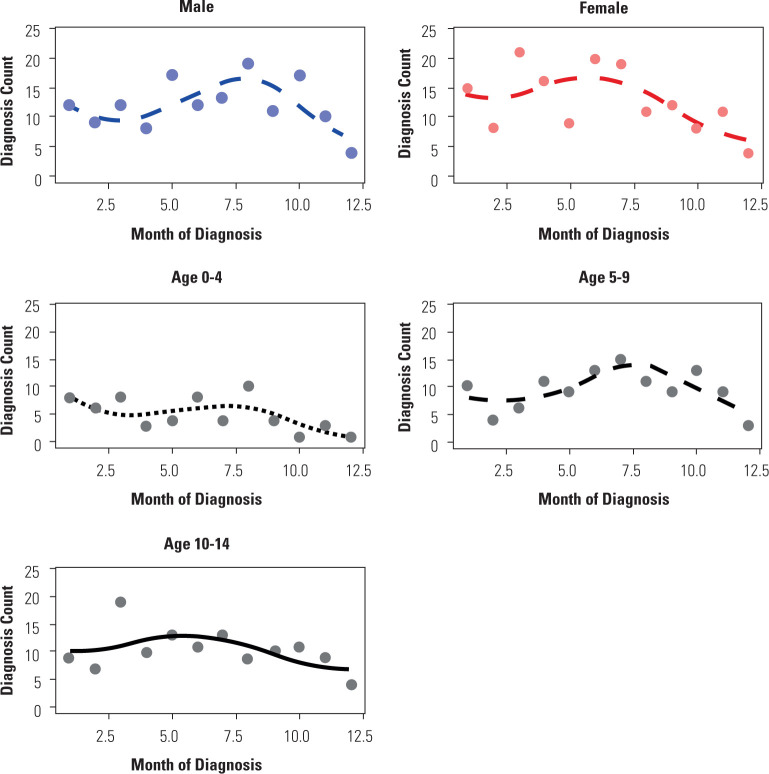
Seasonal variations by sex and age-group, with monthly counts aggregated over the 32-yr period.

Seasonal peaks were noted during the colder months, primarily in winter (June–August), as shown in **Figure 3**.

## DISCUSSION

To the best of our knowledge, this is the first South American study to analyze and characterize cyclical and seasonal variation in T1DM incidence. During the study period, the average incidence of T1DM was 12.1 per 100.00 person-years, with an average annual increase of 2.77%. An 18% cyclical variation in T1DM incidence was observed every 7.5 years. Among girls, a recurring 22.7% cycle every 5 years was identified, while no cyclical pattern was found among boys. Seasonal patterns were noted among girls and children aged 5-9.99 years, with higher T1DM counts during colder and drier months.

While we observed a 7.5-year cycle in T1DM incidence, studies from Britain, Italy, and Australia reported cyclical patterns ranging from 4 to 6 years (^2^,^3^,^5^,^6^). However, a Spanish study (1977-2016) found no evidence of cyclicity in T1DM incidence (^16^). These discrepancies may be attributed to regional differences in ancestry-linked genetic profiles, environmental exposures, healthcare infrastructure, diagnostic practices, or temporal variation in immunological factors such as vaccines and viral infections (^2^,^3^,^16^).

An Australian study identified a 5-year cyclical pattern for both boys and girls (^5^). Conversely, studies from Italy and Scotland reported cyclical patterns only for boys, whereas our findings revealed a 5-year cycle only among girls (^3^,^17^). By contrast, a Spanish and a multicentric European study did not find sex-related differences in T1DM cyclicity (^4^,^16^). These inconsistencies may reflect the complex interplay between hormonal fluctuations and immune system dynamics, which vary significantly by sex (^3^,^5^,^16^). Estrogen and testosterone are known to modulate immune responses and may influence the onset of autoimmune conditions (^3^,^5^,^16^). Additionally, X-linked genetic factors – including immune-related genes on the X chromosome – may contribute to susceptibility and potentially shape the duration or intensity of cyclical trends (^4^,^17^).

This study found seasonal patterns among children aged 5-9.99 years. However, a 5-year pattern in T1DM cyclicity was identified across all age groups in an Australian study (^5^), while an English study reported T1DM cyclical patterns only among children older than 10 years of age (^2^). Similarly, a British study described T1DM cyclicity in children older than 5 years (^6^). These differences may be attributed to variations in immune system maturation, which could influence the susceptibility to autoimmune processes and environmental triggers (^2^,^5^,^6^). Age-related behavioral and physiological factors – such as increased exposure to seasonal infections or fluctuations in metabolic demands – may also influence the timing and intensity of T1DM cyclical and seasonal patterns (^2^,^5^,^6^).

Studies frequently report T1DM cycles aligning with the coldest and driest months (^2^-^6^). This may be due to increased incidence of infections and greater temperature fluctuations during these periods (^2^-^6^).

Several European studies suggest that certain infectious agents – such as influenza, cholera, plague, measles, and mumps – display cyclical epidemics that often correspond with T1DM incidence cycles (^2^,^6^). These studies also indicate that colder months in the Northern Hemisphere, from October to March, are associated with T1DM incidence cycles (^2^,^6^). In Bauru, the current study site, H1N1 outbreaks occurred in 2009, along with pertussis in 2012 and dengue epidemics in 2011 and 2013 (^18^-^20^). Additionally, the lowest mean temperatures were recorded in 2004, 2011, and 2013, according to the local meteorological agency (^21^). Coincidentally, 2009, 2011, and 2013 – years marked by H1N1 and dengue outbreaks and low temperatures –presented higher T1DM incidence rates, supporting the 7.5-year cyclical pattern observed in this study.

This study has notable strengths in methodological design and comprehensive data coverage. As a retrospective longitudinal study spanning over three decades, it provides a robust historical perspective on T1DM trends in Bauru. By integrating a curated local database and combined analytical techniques, this study contributes with valuable findings to the understanding of T1DM epidemiology, aiding future public health planning and intervention strategies (^22^).

Nonetheless, several limitations should be acknowledged. First, the years after 2017 were excluded due to incomplete data, and the confounding effects of dengue and COVID-19 pandemics. This exclusion limited the study’s ability to present a more current overview of incidence trends. Second, despite the formation of a broad task force, underreporting of T1DM may have occurred. Such underreporting could introduce bias in the analyses of long-term disease trends, especially in recent years.

In conclusion cyclical variations in T1DM following a 7.5-year cycle were observed, with girls showing a pronounced variation and a distinct 5-year cycle. Seasonal patterns were evident, with greater amplitude among girls compared to boys, and with the highest seasonal amplitude noted in the 5-9.99-year age group. Outbreaks of H1N1 and dengue, along with the lowest temperatures, coincided with higher T1DM incidence, aligning with the 7.5-year cyclicity observed. These findings suggest that sex, age, viral infections, and temperature may be associated with cyclical and seasonal T1DM incidence patterns, indicating a potential role for environmental and biological triggers. Future studies are warranted to confirm our findings. Targeted health policies are needed to mitigate the impact of these factors, helping optimize the surveillance, early diagnosis, and targeted prevention strategies for this disease.

## Data Availability

datasets related to this article will be available upon reasonable to the corresponding author.
